# Anti-Oxidative and Immune Regulatory Responses of THP-1 and PBMC to Pulsed EMF Are Field-Strength Dependent

**DOI:** 10.3390/ijerph18189519

**Published:** 2021-09-09

**Authors:** Silvia Groiss, Roland Lammegger, Dagmar Brislinger

**Affiliations:** 1 Division of Cell Biology, Histology and Embryology, Gottfried Schatz Research Center, Medical University of Graz, 8010 Graz, Austria; silvia.groiss@medunigraz.at; 2 Institute of Experimental Physics, Graz University of Technology, 8010 Graz, Austria; roland.lammegger@tugraz.at

**Keywords:** extremely low frequency pulsed electromagnetic fields (ELF-PEMF), reactive oxygen species (ROS), EM field strength, anti-oxidative response, immune stimulation

## Abstract

Innate immune cells react to electromagnetic fields (EMF) by generating reactive oxygen species (ROS), crucial intracellular messengers. Discrepancies in applied parameters of EMF studies, e.g., flux densities, complicate direct comparison of downstream anti-oxidative responses and immune regulatory signaling. We therefore compared the impact of different EMF flux densities in human leukemic THP1 cells and peripheral blood mononuclear cells (PBMC) of healthy donors to additionally consider a potential disparate receptivity based on medical origin. ROS levels increased in THP1 cells stimulated with lipopolysaccharide (LPS) after one hour of EMF exposure. Moreover, weak EMF mitigated the depletion of the reducing agent NAD(P)H in THP1. Neither of these effects occurred in PBMC. Landscaping transcriptional responses to varied EMF revealed elevation of the anti-oxidative enzymes PRDX6 (2-fold) and DHCR24 (6-fold) in THP1, implying involvement in lipid metabolism. Furthermore, our study confirmed anti-inflammatory effects of EMF by 6-fold increased expression of IL10. Strikingly, THP1 responded to weak EMF, while PBMC were primarily affected by strong EMF, yet with severe cellular stress and enhanced rates of apoptosis, indicated by HSP70 and caspase 3 (CASP3). Taken together, our results emphasize an altered susceptibility of immune cells of different origin and associate EMF-related effects with anti-inflammatory signaling and lipid metabolism.

## 1. Introduction

Reports on the sensitivity of human health to electromagnetic fields (EMF) have accumulated around the globe and manifest clinically as electromagnetic hypersensitivity (EHS) with broad-spectrum symptoms in multiple organs [1,2,3]. The European Union acknowledged and tackled EHS concerns in the Directive 2013/35/EU, recommending exposure levels of maximum 0.1 mT for public exposure, up to 1 mT for workers at particular risk (WPR), and accepts levels of 3–6 mT in temporary situations [4,5]. Similarly to WPR, medically preconditioned individuals were suggested to be more susceptible to EHS as their survival rate was found to decrease under EMF exposure [6]. Especially high current power lines, such as railway electrification operating at 16.7 Hz, raised concerns claiming increased risk for childhood leukemia [7,8,9,10]. Pinpointing the molecular causality for symptoms accounted to EHS to be indeed caused by EMF is complicated by the complex human system responding to the fluctuating factors of the modern environment that it is exposed to on a daily basis, such as (viral) infections, medication and psychological factors like stress, but also the wide range of frequencies and field strengths emitted from different devices (e.g., smartphones, TV, WiFi, radio) [11]. In parallel, disparities in exposure conditions, i.e., field strength, are also present in in vitro studies focused on EMF-induced effects, which renders direct comparison difficult. For example, while in vitro studies on the leukemic cell line THP1 reported no effects at exposure levels equivalent to the geomagnetic field of <50 µT, exposure to strong EMF of 1 mT seemed to protect THP1 from oxidative stress induced by lipopolysaccharides (LPS), suggesting a gradual receptivity of cells to such physical stimuli [12,13]. Other publications also reported diverging outcomes on cellular receptivity and mechanistic implication when employing different field strengths that range from moderate EMF emitted from power lines < 300 μT [14,15,16], to strong EMF of mT magnitude [17,18].

Nevertheless, at this stage, most studies agree that EMF-induced hyper-production of reactive oxygen species (ROS) is the main cause for EHS [19,20,21,22]. This is not surprising as ROS mediate a myriad of cellular responses of both beneficial and harmful nature [23,24]. ROS are generated upon stimulation by external stimuli such as LPS via oxidation of reducing agents like NAD(P)H, potentially pointing to the availability of such agents as a critical factor for EMF receptivity [25,26]. Excessive levels of cellular ROS are met by a cascade of anti-oxidative enzymes such as superoxide dismutases (SODs), glutathione peroxidases (GPXs), catalase (CAT) and peroxiredoxins (PRDXs). Hence, their availability may be crucial to a cell’s capability to manage oxidative stress [27].

ROS are also crucial second messengers for immune activation [28]. Indeed, increasing evidence suggests that EMF mitigates expression of pro-inflammatory cytokines, e.g., interleukin 1β (IL1β) or tumor necrosis factor α (TNFα), while stimulating levels of interleukin 10 (IL10), a cytokine with strong anti-inflammatory activity [13,29,30]. Since balancing pro- and anti-inflammatory signaling is imperative in tissue regeneration, EMF application holds the potential for a novel non-invasive and non-pharmaceutical treatment option, especially for chronic wounds [29,31].

In order to prevent adverse health effects of EMF in WPR and medically preconditioned patients on the one hand, while effectively applying EMF for immune modulation on the other, it is essential to comprehensively understand the exposure conditions such as flux density, frequency or exposure duration, at which EMF induces the desired effect.

Hence, we aimed to investigate the cellular susceptibility of activated immune cells to EMF based on exposure level and health condition hypothesizing to confirm a strict dependency between field strength and the cell’s health status that would assist guidelines for future applicability of EMF. In this study, we stimulated leukemic THP1 cells in comparison to peripheral blood mononuclear cells (PBMC) derived from healthy individuals with LPS for activation, and exposed them to an EMF equivalent of 16.7 Hz at different flux densities of <50 µT (termed weak EMF in this study; wkEMF), <250 μT (moderate EMF; mdEMF) and <4.8 mT (strong EMF; stEMF). We first assessed stEMF-induced ROS generation on a single cell level using our expertise on automated image analysis [32] and evaluated cellular NAD(P)H levels. Next, we screened for transcriptional anti-oxidative and immune regulatory responses of which key hits were probed in detail for field strength dependent activation, alongside typical key players in anti-oxidative defense and pro- and anti-inflammatory signaling.

Our results clearly indicate disparate receptivity to EMF in immune cells of different medical origin, thereby encouraging further studies to narrow down on parameters relevant for both safe and efficient application of EMF in therapy.

## 2. Materials and Methods

### 2.1. EMF Field Strength Verification and Cellular Exposure

**Figure 1:**
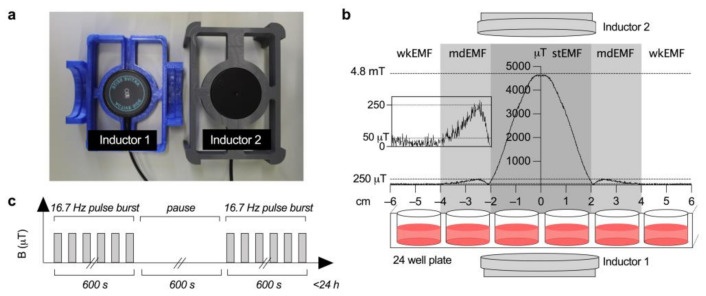
Setup and parameter for EMF exposure. (**a**) Culture plate holder for exact positioning of both inductors towards the sample plate. (**b**) Differences in EMF field strength at the individual sample positions of the culture plate for exposure of wkEMF (<50 μT), mdEMF (<250 μT, close up view shown in insert) and stEMF (<4.8 mT). (**c**) A fundamental exposure frequency of 16.7 Hz is applied using a rectangular wave form for up to 24 h with send/pause intervals of 600 s.

The EMF was generated using the IMEDIS MINI-EXPERT-T system (provided by IPP-Ing. Peter Peutler GmbH, Graz, Austria). Two identical inductors (#1 located vertically above #2 and the sample, cylindrical dimensions Ø 55 × 22.5 mm) were positioned in a specifically designed plate holder to ensure precise orientation, at a clear distance of 11 mm from the facing inner surfaces of the sample area (Figure 1a). The plastic housing holds the actual ring coils with diameters of 20 mm (inner) and 39 mm (outer). Each ring coil has 2700 turns of copper wire with a wire diameter of 0.25 mm. The two inductor Helmholtz-like arrangement achieves coil-factors (B-field/coil current) of 124 µT/mA (referenced to the center plane that is 5.5 mm away from the inner surfaces) and 223 µT/mA (referenced to the sample plane; both at x = y = 0.0 mm; identical to the center plane). The positive *z*-axis of the reference system is ultimately important and coincides with the rotation axis of the inductors pointing vertically upwards. The sample plane (with the culture plates) lies 3.0 mm below the center plane (z = −3.0 mm).

The magnetic flux density (B-field) at the respective locations of the culture plate (sample plane) was determined with a two-component Hall sensor by measuring the two B-field components in the direction of the coil axis (vertical) and transverse (horizontal). The spatial integration volume of the sensor is about 0.5 mm^3^ and the accuracy of the B-field magnitude is about 1%. The position of the sensor was determined with two rotary encoders (524082 from Leybold Didactics) at an accuracy of ±0.1 mm.

The course of the magnetic field in the sample plane is typical for this configuration with a homogeneous B-field curve (approx. constant in z-direction over the samples), steadily decreasing in the XY plane up to approximately the edge of the inductors, where the z-component of the B-field reverses due to the B-field’s vortex field property. This is represented as a renewed increase of the B-field’s scalar value in the range of x = ± 2 to ±4 cm (mdEMF; Figure 1b). In the outermost region x = ± 4 to ± 6 cm (wkEMF), the B-field approaches the laboratory ambient magnetic field of approximately 40 µT. It has to be mentioned that an additional AC magnetic field stemming from power line interferences of about 0.2 µT_eff_ (mainly 50 Hz and the odd harmonics) is present in the laboratory.

For EMF application experiments, both THP1 and PBMC were stimulated with 1 μg/mL LPS from Escherichia coli O111:B4 (Sigma-Aldrich, St. Lois, MO, USA), a well-known trigger of ROS-mediated immune signaling [28,33], and immediately exposed to a pulsed EMF (referred to as EMF) of either <50 μT (wkEMF), <250 μT (mdEMF) or <4.8 mT (stEMF, B-field at z-position of cells; Figure 1b) with a fundamental frequency of 16.7 Hz emitted in send/pause intervals of 10 min (on/off; Figure 1c) for up to 24 h with sampling after 1 h, 3 h, 6 h and 24 h. Control cells were stimulated with 1 μg/mL LPS and positioned in an identically constructed incubator without application of EMF. All experiments were performed at 37 °C and 5% CO_2_.

### 2.2. Primary Cells and Cell Lines

The human monocytic cell line THP1, derived from an acute monocytic leukemia patient, were purchased from ATCC (American Type Culture Collection, Nr. TIB-202, Wesel, Germany) and maintained in RPMI-1640 media (Sigma-Aldrich) supplemented with 10% FBS (PAA Laboratories GmbH), 20 mM HEPES (Sigma-Aldrich) and 100 μM penicillin/streptomycin (P/S, Sigma-Aldrich) at a density of 0.2 to 1 × 10^6^ cells/mL. Human PBMCs were purchased from STEMCELL Technologies (Cologne, Germany, Cat. #70025) and used immediately after thawing as instructed by the manufacturer. All cells were maintained at 37 °C at 5% CO_2_. For EMF experiments, both THP1 and PBMC were maintained in RPMI-1640 media supplemented with 10% human serum, 20 mM HEPES and 100 μM P/S at 37 °C at 5% CO_2_.

### 2.3. Detection of Superoxide Levels Using Dihydroethidium

Total superoxide (O_2_^−^) levels were assessed using dihydroethidium (DHE; D7008, Sigma-Aldrich). Samples were taken at the respective time point, incubated with DHE at a concentration of 2 µM for 30 min at 37 °C, washed 3× with PBS, transferred to SuperFrost Plus™ slides (Thermo Fisher Scientific, Waltham, MA, USA) by cytospin (80 rpm for 5 min at room temperature (RT); Shandon Cytospin II, GMI) and left to dry for 1 h at RT. After fixation in acetone for 10 min, the cells were rehydrated in PBS, counterstained using 4′,6-diamidino-2-phenylindole (DAPI) for 10 min at RT, dehydrated in a graded series of ethanol (70%, 96% and 100%) and dried for 5 min before mounting with ProLong™ Gold Antifade Reagent (Thermo Fisher Scientific).

### 2.4. Image Acquisition and Computational Analysis

**Figure 2:**
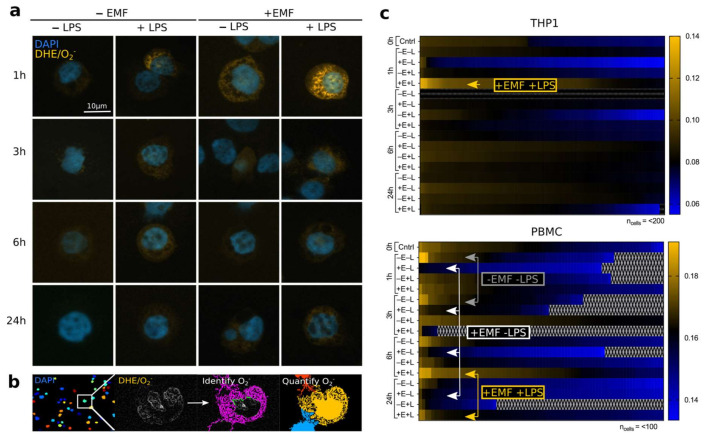
stEMF-modifies O_2_^−^ levels in THP1 and PBMC. THP1 and PBMC with and without LPS stimulation were exposed to stEMF (<4.8 mT) for 1 h, 3 h, 6 h and 24 h, incubated with DHE for O_2_^−^ detection and transferred to a slide by cytospin for DAPI staining and imaging. Representative single cell images of THP1 cells are shown in (**a**) for the tested exposure times and conditions. Fluorescence levels of DHE were detected and quantified using a specifically devised Cell Profiler pipeline shown in (**b**). (**c**) Increased O_2_^−^ levels in LPS stimulated and EMF exposed samples are indicated in yellow (THP1 and PBMC). In PBMC, EMF exposure without LPS stimulation seemed to mitigate O_2_^−^ formation (white arrows); basal levels at 1 h and 3 h are highlighted in gray. *N* = 200 (THP1) or *n* = 50–100 (PBMC) single cells were analyzed per individual sample.

The fluorescence intensity of O_2_^−^ staining by DHE was captured using a Zeiss Axio Observer Z1 Inverted Microscope equipped with a 120 W HXP Mercury short-arc lamp and an Axiocam 702 mono camera equipped with an excitation and emission filter set for visualization of FITC, Cy3, Cy5 and DAPI using a 40× (LD Achroplan 40×/0.60 corr., D = 0–2 mm) objective with the ZEN 2 (blue) software (Version 2.0.0.04.8.2.0; all Carl Zeiss, Oberkochen, Germany). Nine adjacent images in original gray-scale were taken in tile mode covering a total of 50–200 cells per sample and time point. Brightness and contrast of representative single cells were modified for enhanced visualization only (Figure 2a). Data analysis was performed using the open-source software Cell Profiler (version 3.1.5, [34]). The analysis pipeline was designed to quantify the fluorescence intensity adjacent to the respective nuclei (Figure 2b). Briefly, the module ‘IdentifyPrimaryObjects’ was used to target DAPI staining to assess the total number of cells per image before identifying the adjacent DHE stained area with the module ‘IdentifySecondaryObjects’, whose intensity was calculated by ‘MeasureObjectIntensity’. The data was further processed using Microsoft Excel.

### 2.5. MTT Assay to Assess Level of Reducing Agents

We used a standard 3-(4,5-dimethylthiazol-2-yl)-2,5-diphenyl tetrazolium bromide (MTT) assay (EZ4U Assay, Biomedica, Vienna, Austria; 19, [35]) to assess NAD(P)H levels following manufacturer’s instructions. Although commonly used as a cytotoxicity assay, it measures the reduction of a tetrazolium salt majorly by reducing agents such as NAD(P)H, and can thus be used for their intracellular assessment [36]. The cells were seeded into a 96-well tissue culture plate (Nunc, Thermo Fisher Scientific) at a density of 5 × 10^4^ cells/well, followed by EMF exposure at the respective field strength. At the given time point, the absorbance of dissolved formazan was measured at 584 nm (FLUOstar OPTIMA, BMG Labtechnology, Offenburg, Germany) and is given in percentage of absorbance of the control (non-exposed, non-LPS-stimulated cells).

### 2.6. RNA Isolation, PrimePCR^TM^ and Quantitative RT-PCR Assays

Total RNA was isolated using the peqGOLD Total RNA Kit (Peqlab, Erlangen, Germany) according to manufacturer’s instructions and tested for concentration and integrity using an ND-1000 Spectrophotometer (NanoDrop Technologies, Wilmington, NC, USA). cDNA was synthesized using the RevertAid First Strand cDNA Synthesis Kit (Thermo Fisher Scientific) and diluted to 1 ng/µL. Expression levels of anti-oxidative stress (#10034556, PCR array, Bio-Rad, Hercules, CA, USA) and immune regulatory genes (#10025455, Bio-Rad) were screened after 3 h of stEMF exposure. PrimePCR™ key hits alongside additional relevant anti-oxidative and immune regulatory targets were further analyzed at the respective field strength by standard quantitative real time PCR (RT-PCR) using a CFX384™ Real-Time System (Bio-Rad) and the iTaq™ Universal SYBR^®^ Green Supermix (Bio-Rad). Primers were purchased from Microsynth (Vienna, Austria) and are shown in Supplemental Table S1. The following cycle program was used: 95 °C for 3 min initial denaturation, 95 °C for 10 s, 58 °C for 30 s and 72 °C for 30 s, repeated 39x followed by melting curve analysis (60–90 °C in 0.5 °C/30 s increments). Gene expression levels were normalized against ß-actin. Relative expression levels were calculated by the standard 2^−∆∆Ct^ method, correlated to the mean intensity of cells at t0 and normalized to the LPS stimulated control at the given time point.

### 2.7. Statistics

Statistical analysis and data representation was performed using GraphPad Prism version 9 (GraphPad Software, www.graphpad.com accessed on 19 July 2021). Data are presented as box blots or bar diagrams (mean ± SEM) of at least three independent experiments measured at technical duplicates (*N* ≥ 3, *n* = 2). Differences between groups were assessed by two-way ANOVA followed by Dunnett’s test for multiple comparison with significance levels at *p* < 0.05 (*), *p* < 0.01 (**), *p* < 0.001 (***) and *p* < 0.0001 (****). *p* < 0.08 (#) was considered as trend.

## 3. Results

### 3.1. stEMF Elevates ROS Formation in LPS-Stimulated Cells

EMF-induced cellular ROS levels were assessed by exposing LPS-stimulated THP1 and PBMC to stEMF for 1 h and probed for O_2_^−^ formation on a single cell level (*n* = 200 cells for THP1 and *n* = 50–100 cells for PBMC). We found the strongest increase in O_2_^−^ formation in THP1 cells following LPS stimulation after 1 h of EMF exposure with only minor changes at other time points and with or without stimulation or EMF exposure (yellow arrow). The O_2_^−^ levels of PBMC were strongest after 1 h and 3 h in non-stimulated and non-exposed samples (gray arrows) and after 6 h and 24 h of LPS-stimulated and stEMF-exposed samples (yellow arrows). Surprisingly, EMF exposure without prior LPS stimulation behaved contrarily and seemed to rather abolish O_2_^−^ formation (Figure 2c, white arrows).

These results might suggest that stEMF exposure of LPS stimulated cells boosts LPS-induced ROS formation. Furthermore, the data generally revealed O_2_^−^ to be present in tubular structures potentially allocating the site of formation to the mitochondrial network [37].

### 3.2. wkEMF Mitigates NAD(P)H Depletion in THP1 Cells

**Figure 3:**
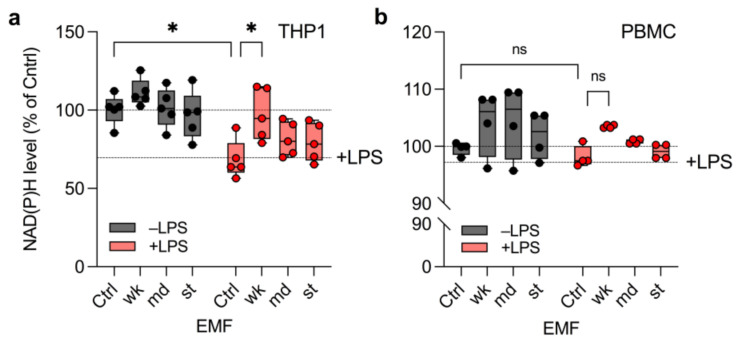
wkEMF counteracts NAD(P)H depletion in LPS-stimulated THP1. Both THP1 (**a**) and PBMC (**b**) were stimulated with or without LPS and exposed to wkEMF, mdEMF or stEMF for 1 h before measuring NAD(P)H levels by MTT assay to assess a potential interference of EMF with LPS-induced NAD(P)H depletion during ROS formation. Data was collected in *n* = 5 (THP1) or *n* = 4 (PBMC) individual experiments. ns: not significant, * *p* < 0.05 as indicated.

Since ROS formation is linked to the cell’s availability of reducing agents such as NAD(P)H [24,26], we tested whether EMF exposure enhances LPS-induced NAD(P)H depletion by exposing THP1 and PBMC to wkEMF, mdEMF and stEMF for 1 h followed by measuring formazan formation using an EZ4U assay. NADP(H) levels declined significantly to 68.4 ± 12.2% upon LPS stimulation in THP1 cells indicating the expected ROS formation (Figure 3a). It was thus highly surprising that wkEMF counteracted the LPS-induced NAD(P)H depletion (97.4 ± 16.6%; Figure 3a). This effect was not observed for mdEMF or stEMF, potentially hinting at a protective effect against NAD(P)H depletion in THP1 only at lower levels of EMF. In comparison, NAD(P)H levels in PBMC neither declined upon LPS stimulation, nor did we observe a differential effect in NAD(P)H levels upon EMF exposure (Figure 3b).

### 3.3. EMF Elevates Expression of Anti-Oxidative Genes Related to Lipid Metabolism

**Figure 4:**
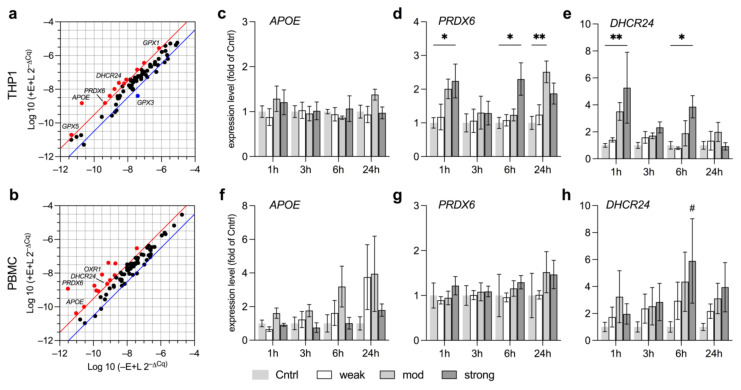
Target screening and evaluation of transcriptional responses of key hits following EMF exposure at varying field strengths. (**a**,**b**) PrimePCR of THP1 (**a**) and PBMC (**b**) exposed to stEMF for 3 h reveals transcriptional deregulation (upregulation, red; downregulation, blue) of several anti-oxidative defense genes (considered if more than 4-fold deregulated). Relevant targets are indicated by name. Three key hits (*APOE*, *PRDX6* and *DHCR24*) representative for both THP1 (**c**–**e**) and PBMC (**f**–**h**) were tested at different time points and field strengths (*N* = 3, *n* = 2). Data are given as fold of Cntrl (mean ± SEM). * *p* < 0.05 and ** *p* < 0.01 as indicated. # *p* < 0.08 representing a trend.

Next, we attempted to identify novel targets in anti-oxidative defense mechanisms that may be elevated upon EMF exposure, and in a second step, assess which exposure levels are required for sufficient induction. Hence, LPS-stimulated THP1 and PBMC were initially exposed to a stEMF for 3 h and screened for deregulation of key anti-oxidative enzymes by PrimePCR array. Selected targets were then evaluated after exposure to wkEMF, mdEMF or stEMF by standard RT-PCR. LPS stimulated and non-exposed cells were used as controls. Targets showing more than 4-fold deregulation in THP1 (Figure 4a) and PBMC (Figure 4b) were considered relevant and are marked in red (upregulated) or blue (downregulated). The major deregulated genes in THP1 and PBMC are given in Supplemental Table S2 including the coherent fold change. It was highly interesting that among genes typical for the degradation and regulation of ROS, such as glutathione peroxidases (*GPX*, [38]; *GPX1* and *GPX5* elevated in THP1) or oxidation resistance 1 (*OXR1*, [39]; elevated in PBMC), we found expression levels of genes relevant in the lipid metabolism such as apolipoprotein E (*APOE*, [40,41]), peroxiredoxin 6 (*PRDX6*, [42,43]) and 24-dehydrocholesterol reductase (*DHCR24*, [44]) to be increased in both THP1 and PBMC (Figure 4a,b). We chose these three as the key hits to investigate whether their induction is subject to the exposure level by probing their expression at several time points following exposure to wkEMF, mdEMF or stEMF. While we found no correlation of the field strength of EMF on *APOE* expression, levels of *PRDX6* and *DHCR24* were significantly increased after exposure to stEMF in THP1 cells but not PBMC (Figure 4c–h).

Clearly, EMF in the mT range seemed to interfere with cholesterol synthesis and/or low-density-lipoprotein (LDL) oxidation, thus warranting further investigations for potential application of EMF in lipid metabolic disorders.

### 3.4. Anti-Oxidative Responses in THP1 and PBMC Vary by Field Strength

**Figure 5:**
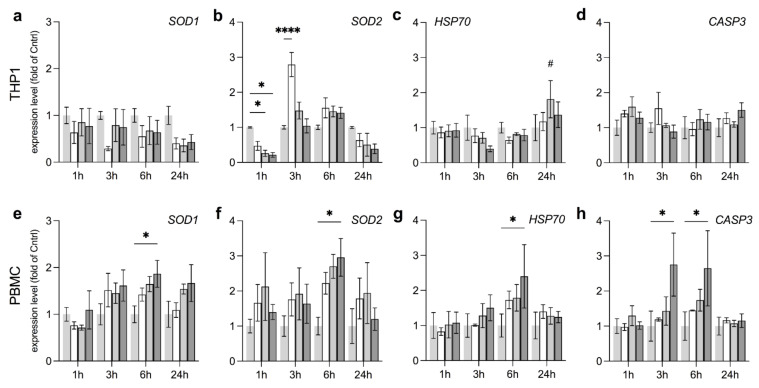
THP1 shows a pronounced anti-oxidative effect at wkEMF, while stress and pro-apoptotic activity was induced in PBMC only at stEMF. To investigate a wider range of cellular responses to different EMF exposure levels, we probed the expression levels of the key anti-oxidative enzymes *SOD1* and *SOD2*, as well as the stress and pro-apoptotic enzymes *HSP70* and *CASP3*, in (**a**–**d**) THP1 and (**e**–**h**) PBMC at different field strengths. Data are given as fold of Cntrl (mean ± SEM, *N* = 3, *n* = 2). * *p* < 0.05 and **** *p* < 0.0001 as indicated. # *p* < 0.08 representing a trend.

The enzymes SODs (i.e., SOD1 and SOD2), GPXs and CAT are at the forefront of ROS degradation, among others. We therefore additionally tested these targets alongside heat shock protein 70 (HSP70) and caspase 3 (CASP3) to assess overall levels of cellular stress and rates of apoptosis as a potential marker for EMF-induced cytotoxicity at different field strengths. In THP1, EMF progressively reduced SOD1 over time, impartial of the exposure level as can be seen from the 24 h time point (Figure 5a). In contrast, SOD2 was substantially reduced already after 1 h, especially when exposed to mdEMF or stEMF (4 to 5-fold reduction, respectively) but significantly increased again after 3 h of exposure, which is best represented by a 2.8-fold elevation at wkEMF, as compared to the control (Figure 5b). This pattern, together with reduced rates of NAD(P)H depletion in THP1 cells (Figure 3a) suggests that EMF exposure halts and subsequently amplifies ROS formation, rather than a direct immediate induction. Additionally, the increase in SOD2 expression supports the generation of ROS to take place primarily within the mitochondrial network as already implied by DHE detection within tubular structures (Figure 2). GPX1 was elevated after 3 h in stEMF with no changes in CAT expression (Supplemental Figure S1a,b). Although we found increased levels of HSP70 at mdEMF at 24 h, no changes in CASP3 were detected, overall neglecting pro-apoptotic effects of EMF in THP1 (Figure 5c,d). In contrast, SOD1 and SOD2 levels increased in PBMC after 6 h of exposure to stEMF without elevation of GPX1 or CAT (Figure 5e,f and Supplemental Figure S1c,d). Furthermore, stEMF significantly increased levels of HSP70 and CASP3 when compared to LPS-stimulated controls (Figure 5g,h).

Overall, these findings suggest that while EMF increases oxidative stress in THP1 at lower exposure levels without displaying pro-apoptotic activity, PBMC respond only to stEMF, yet here with great cellular stress and increased rates of apoptosis.

### 3.5. EMF Increases Pro-Survival Factors in THP1

**Figure 6:**
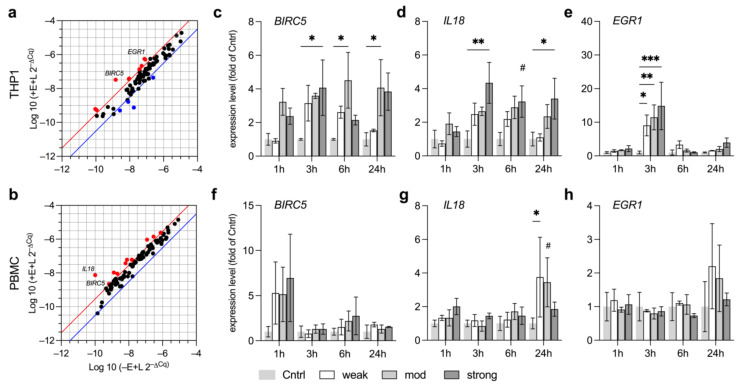
PrimePCR and target expression analysis of key immune regulatory genes following exposure to EMF at different field strengths. (**a**,**b**) Prime PCR of immune disease related targets probing THP1 (**a**) and PBMC (**b**) after exposure to stEMF for 3 h. Genes were considered as upregulated (red) or downregulated (blue) if deregulated more than 4-fold compared to the LPS-stimulated and non-exposed control. The key hits *BIRC5*, *IL18* and *EGR1* (indicated by name) that are elevated in the PrimePCR of either THP1 (**c**–**e**) and/or PBMC (**f**–**h**) were tested at different time points and field strengths (*N* = 3, *n* = 2). Data are given as fold of Cntrl (mean ± SEM). * *p* < 0.05, ** *p* < 0.01 and *** *p* < 0.001 as indicated. # *p* < 0.08 representing a trend.

Having determined the expression levels of certain anti-oxidative enzymes, we then aimed to detail the immunological landscape influenced by EMF exposure at the transcriptional level. We screened for deregulated genes by PrimePCR array before testing key targets at different field strengths to additionally assess required exposure levels of such stimulation. Deregulated genes showing more than 4-fold changes are shown in Figure 6a (THP1) and Figure 6b (PBMC; upregulation, red; downregulation, blue). Genes and their differing regulations are given in Supplemental Table S3.

Among the top hits upregulated in THP1, and to a minor degree in PBMC, was the baculoviral inhibitor of apoptosis repeat-containing 5 (BIRC5). BIRC5 is a well-known biomarker for tumor progression as it promotes cell proliferation and overall rates of survival and is associated with poor clinical outcome in patients with acute myeloid leukemia (AML) [45,46]. THP1 cells are derived from a patient with AML and are therefore expected to show high basal levels of BIRC5. Still, mdEMF and stEMF significantly elevated *BIRC5* at several time points (on average by 4-fold) when compared to LPS-stimulated and non-exposed controls, thereby further strengthening pro-survival signaling in THP1 (Figure 6c). stEMF further upregulated the pro-inflammatory cytokine interleukin 18 (*IL18*) alongside early growth response 1 (*EGR1*), an important transcription factor with multifaceted roles in tumor formation and proliferation as well as inflammation ([47,48], Figure 6d,e). The drastic increase of *EGR1* even at wkEMF exposure measured at the 3 h time point, in particular, highlights the severe scope of potential downstream-regulated pathways and hence the breadth of potential responses induced even by wkEMF. Neither *BIRC5* nor *EGR1* were significantly elevated in PBMC, while *IL18* was increased after 24 h only at wkEMF, overall neglecting a potential proliferative or major pro-inflammatory effect of EMF in PBMC (Figure 6f–h).

### 3.6. stEMF Elevate Anti-Inflammatory Signaling in THP1 but Not PBMC

**Figure 7:**
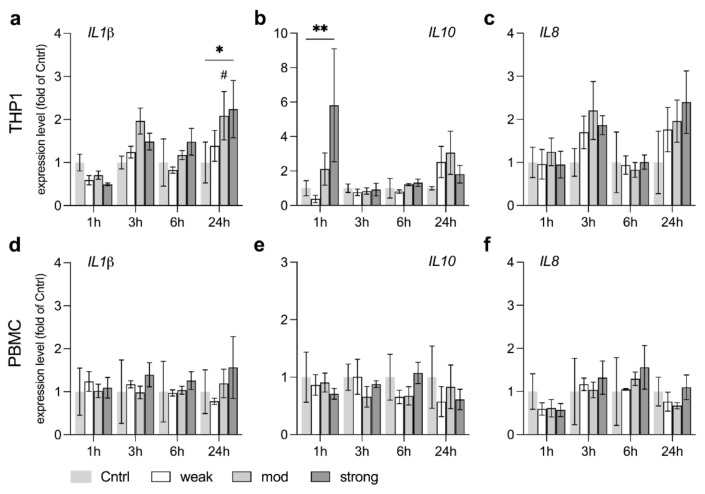
stEMF exposure increases expression of inflammatory cytokines in THP1 (**a**–**c**) but not PBMC (**d**–**f**). Expression levels of the major pro- and anti-inflammatory cytokines *IL1B* (**a**,**d**) and *IL10* (**b**,**e**), as well as the chemokine *IL8* (**c**,**f**), were tested after exposure to wkEMF, mdEMF or stEMF to investigate potential immune stimulating effects of EMF in a dose-dependent approach. Data are given as fold of Cntrl (mean ± SEM, *N* = 3, *n* = 2). * *p* < 0.05 and ** *p* < 0.01 as indicated. *p* < 0.08 (#) indicates a trend.

Pro- and anti-inflammatory cytokines act in concert to balance the complex interplay of immune responses and maintain organism homeostasis [49]. We therefore tested the key pro- and anti-inflammatory cytokines *IL1B* and *IL10*, as well as the chemokine *IL8*, in their transcriptional regulation upon EMF exposure in LPS stimulated cells [49,50]. While *IL1B* levels increased after 24 h in stEMF exposed THP1, *IL10* levels were already 6-fold elevated after 1 h of exposure, suggesting anti-inflammatory rather than pro-inflammatory signaling as immediate responses induced by EMF (Figure 7a,b), which is in line with previous studies [13]. No significant elevation in *IL1B* or *IL10* was observed in PBMC (Figure 7d,e). IL8 levels remained stable in both THP1 and PBMC across all exposure levels and time points (Figure 7c,f).

## 4. Discussion

The rampant and permanent increase in both public and occupational exposure to various EMF fuels ongoing discussion on health concerns, with the recent implementation of 5G as the most prominent example [51,52]. The complexity of such physical stimuli, in particular, necessitates considering the applied conditions such as frequency and field strength, as well as the fundamental medical condition of the individual. While some studies tested variances in the exposure frequency [15,53], only few compared different field strengths. Van Huizen and colleagues reported compelling differences on stem cell proliferation at highly distinct field strengths, and emphasized the importance of accurately defining such parameters when used for tissue regeneration [54]. This might indicate that biological systems respond to very precise physical stimuli while remaining inert to others.

Inspired by these results, we aimed to further detail the applied parameters required to induce cellular responses to EMF by exposing immune cells of different origin to varied field strengths. Our initial data on ROS formation revealed higher basal levels in LPS-stimulated PBMC than in THP1, with negligible increments upon EMF stimulation. PBMC comprise a subset of lymphocytes, monocytes, and dendritic cells. As monocytes represent a minor subpopulation, increased ROS formation in LPS-stimulated PBMC is therefore probably a consequence of stimulated lymphocytes rather than of stimulated monocytes. However, ROS levels increased in THP1 after 1 h of exposure, which is in line with other studies on THP1 [55,56]**,** as well as various other cell types [20,57,58]. It was thus surprising that wkEMF without prior LPS stimulation seemed to diminish ROS levels in PBMC. Yet, a protective effect of repeated EMF stimulation against oxidative stress was also reported in human neuroblastoma SH-SY5Y cells challenged with H_2_O_2_ following EMF exposure [59], which supports hypotheses on improved anti-oxidative stress responses upon EMF treatment [20].

Since ROS formation exploits cellular NAD(P)H levels [26,27], a reduced decay of NAD(P)H in THP1 upon the exposure to wkEMF, but not mdEMF and stEMF, was unexpected. Hence, the response of THP1 was greatest when exposed to weak EMF around 50 µT. It is possible that EMF exposure disrupts ROS formation leading to a subsequent boost, which is further displayed by the reduction of *SOD2* at 1 h followed by a significant elevation at the 3 h time point. Although effects of low magnitude EMF on THP1 are in disagreement with Bouwens et al., who reported no effect on THP1 if exposed to EMF as low as 5 µT [12], other studies also spotted cellular receptivity to EMF at similar exposure levels to ours [54,57]. Considering that 50 µT, which is in the range of the geomagnetic field, was the lowest exposure level used in our study, 5 µT was likely too low an EMF to have a noticeable effect, which is in line with the majority of studies reporting effects of EMF on THP1 at 1 mT field strength [25,60,61].

Similarly, stEMF of <4.8 mT induced expression of *PRDX6* and *DHCR24*, enzymes involved in lipid metabolism, in THP1 in our study, which was not observed for exposure levels <250 µT. These findings compare well to previous studies reporting lipid peroxidation induced by EMF at mT exposure levels [62]. Furthermore, static EMF were recently shown to treat type 2 diabetes in mice [63], which together with our findings might widen the portfolio of potential applications of EMF in lipid metabolic disorders such as atherosclerosis [42,44,64]. PBMC showed a trend towards upregulation of *DHCR24* after exposure to stEMF that was complemented by elevated expression levels of *SOD1* and *SOD2*. The increase in *SOD2*, as the respective dismutase in the mitochondrial matrix, is not only in line with other studies reporting *SOD2* upregulation upon EMF exposure [62], but also confirms the site of ROS formation to be majorly situated within the mitochondrial network [65].

It is further suggested that susceptibility to EMF is influenced by the fundamental medical condition [4,6]. This was also true when translated to in vitro experiments, in which only osteoblasts with an initial low capacity for matrix mineralization responded to EMF treatment [53]. These results were highly surprising considering that the beneficial effects of EMF for fracture healing or osteoporosis were already approved by the FDA in the 1980s and are widely accepted in the clinical setting for several applications [66,67,68,69].

We observed similar disparities in our study. Expression of *HSP70* and *CASP3*, important markers for cellular stress and pro-apoptotic activity, remained rather stable in THP1, despite the induction of oxidative stress at wkEMF as indicated by *SOD2* levels. Quite to the contrary, PBMC displayed strong induction of *HSP70* and *CASP3* at stEMF, pointing towards increased cellular toxicity of EMF in PBMC at mT magnitude, as previously reported [70]. Increased levels of *HSP70* at exposure levels of 4 mT were also shown in flying insects and even report the flies to be physically impaired in walking [71]. Furthermore, it is of concern that our study reports *BIRC5* levels to strongly increase in THP1 exposed to mdEMF and stEMF at several time points. Perhaps our data already constitute a potential mechanistic explanation to reports on increased childhood leukemia upon residential EMF exposure, as BIRC5 promotes cell proliferation and constitutes an important biomarker to predict the clinical outcome in AML patients [45]. Similarly, EMF upregulated EGR1 expression, a multifaceted transcription factor that mediates proliferation but also inflammatory signaling [47,48], constituting another potential link to observed effects on inflammation.

In strong contrast, previous reports also emphasized the protective role of EMF against pathogenic stimuli such as LPS by primarily elevating anti-inflammatory cytokines while repressing pro-inflammatory cytokines, e.g., IL10 vs. IL1β or TNFα, which is in line with our findings. These results strengthen the role of EMF for potential application in tissue repair [13,29]. Costin and colleagues nicely reviewed the extent to which these effects translate to in vivo, and report that cytokine profiles transitioned to the anti-inflammatory state required for wound closure especially in chronic ulceration, among other beneficial effects for regenerative therapy.

Clearly, the effects observed in our study are not directly transferable considering that we measured transcriptional levels, which cannot be translated directly to the protein level or even the activity of certain enzymes. However, studies show that RNA levels correlated better to their protein equivalent if differentially expressed under experimental stimulation [72], as is the case in our study. Moreover, we chose to measure RNA levels as these might be more suitable to clarify the cell’s immediate response, hence demonstrating how a certain EMF is “perceived” by the cell rather than how cells “act” (on a protein level).

Nevertheless, our results pilot findings that immune cells of pathologic background may be affected by lower field strengths than cells of healthy background, encouraging risk stratification in health surveillance guidelines. Furthermore, these findings corroborate the induction of cellular stress and pro-apoptotic activity in cells of healthy origin when exposed to EMF at mT magnitude, and for the first time indicate a potential mechanistic link to EMF-related reports on leukemia through elevated expression of *BIRC5*. On the other hand, we observed cellular responses that confirm the application of EMF for immune system stimulation and even identify potential novel targets for the treatment of lipid metabolic disorders by EMF therapy.

In any case, more extensive and orthogonal studies on field strength and host susceptibility are called for. Future goals in EMF research must therefore further define parameters that shift the observed harmful effects of EMF towards their favorable application in more detail. In this way, EMF might enable a diverse range of novel non-invasive and non-pharmaceutical therapies as nicely exemplified by the FDA-approved EMF therapy in fracture healing.

## 5. Conclusions

Taken together, our results illustrate different susceptibilities to EMF in innate immune cells of different origin and highlight discrepancies in the governing field-strength. While EMF promoted anti-oxidative defense and anti-inflammatory signaling, increased rates of cellular stress and apoptosis indicate caution at exposure levels in the mT range. However, EMF holds strong potential for application in a myriad of diseases, especially in tissue regeneration, under the caveat that key parameters, such as health status of the patient and applied frequency and field strength, are well understood, further opening up important platforms for discussion and research.

## Data Availability

Data supporting the findings of this study are available from the corresponding author on reasonable request.

## Supplementary Materials

The following are available online at https://www.mdpi.com/article/10.3390/ijerph18189519/s1, Figure S1: Expression levels of GPX1 and CAT in THP1 and PBMC. Table S1. Primer used for RT-PCR. Table S2. Anti-oxidative PrimePCR targets. Table S3. Immune regulatory PrimePCR targets.
