# Supplementary material for: Anti-Oxidative and Immune Regulatory Responses of THP-1 and PBMC to Pulsed EMF Are Field-Strength Dependent
Source: Int J Environ Res Public Health. 2021 Sep 9;18(18):9519. doi: 10.3390/ijerph18189519 (PMC8471206; doi:10.3390/ijerph18189519)
Supplement: Supplementary file 1 [file ijerph-18-09519-s001.zip › ijerph-1352295-supplementary.pdf]

## Supplemental Information

# Anti-oxidative and immune regulatory responses of THP1 and PBMC to pulsed EMF are field-strength dependent

Silvia Groiss [1], Roland Lammegger [2] and Dagmar Brislinger [1,*]

[1] Division of Cell Biology, Histology and Embryology, Gottfried Schatz Research Center, Medical University of Graz, 8010 Graz, Austria; silvia.groiss@medunigraz.at (S.G.),
[2] Institute of Experimental Physics, Graz University of Technology, 8010 Graz, Austria; roland.lammegger@tugraz.at
* Correspondence: dagmar.brislinger@medunigraz.at (D.B.)

Primer sequences used for RT-PCR are given in Table S1.

**Table S1.** Primer used for RT-PCR.

| **Target[1]** | **Forward primer (5' – 3')** | **Reverse primer (5' – 3')** |
|---|---|---|
| *Prime targets* | | |
| *APOE* | GTTGCTGGTCACATTCCTGG | GCAGGTAATCCCAAAAGCGAC |
| *PRDX6* | GTGACAGCTCGTGTGGTGTT | CTGGGGTGGCAACCCTTTT |
| *DHCR24* | CACTGTCTCACTACGTGTCGG | CCAGCCAATGGAGGTCAGC |
| *BIRC5* | AGGACCACCGCATCTCTACAT | AAGTCTGGCTCGTTCTCAGTG |
| *IL18* | TCTTCATTGACCAAGGAAATCGG | TCCGGGGTGCATTATCTCTAC |
| *EGR1* | GGTCAGTGGCCTAGTGAGC | GTGCCGCTGAGTAAATGGGA |
| *Additional targets* | | |
| *SOD1* | GGTGGGCCAAAGGATGAAGAG | CCACAAGCCAAACGACTTCC |
| *SOD2* | GGAAGCCATCAAACGTGACTT | CCCGTTCCTTATTGAAACCAAGC |
| *GPX1* | TGGGCATCAGGAGAACGCCA | GGGGTCGGTCATAAGCGCG |
| *CAT* | TGGAGCTGGTAACCCAGTAGG | CCTTTGCCTTGGAGTATTTGGTA |
| *HSP70* | GCCGAGAAGGACGAGTTTGA | TCCGCTGATGATGGGGTTAC |
| *Casp3* | CATGGAAGCGAATCAATGGACT | CTGTACCAGACCGAGATGTCA |
| *IL1B* | TGAGCTCGCCAGTGAAATGAT | TCCATGGCCACAACAACTGA |
| *IL8* | GAATGGGTTTGCTAGAATGTGATA | CAGACTAGGGTTGCCAGATTTAAC |
| *IL10* | AATAAGGTTTCTCAAGGGGCT | AGAACCAAGACCCAGACATCAA |

[1] All primer were purchased from Microsynth (Balgach, Switzerland).

Data obtained from PrimePCRs on deregulation of anti-oxidative and immune regulatory enzymes/targets is given in Table S 1 and S 2, respectively.

**Table S2.** Anti-oxidative PrimePCR targets with more than 4-fold deregulation in THP1 and PBMC.

| Target | THP1 (fold change) | PBMC (fold change) |
|---|---|---|
| *APOE* | 133.28 | 8.99 |
| *DHCR24* | 13.31 | 8.69 |
| *CYBB* | 10.68 | |
| *LPO* | 7.99 | |
| *NCF1* | 7.69 | 7.96 |
| *GPX5* | 7.25 | |
| *GCLM* | 6.70 | |
| *PTGS2* | 6.49 | |
| *GPX1* | 6.37 | 5.52 |
| *GPX7* | 6.02 | |
| *PRDX6* | 5.55 | 973.99 |
| *FOXM1* | 4.98 | |
| *GSS* | 4.40 | 47.13 |
| *GPX3* | -5.80 | |
| *RNF7* | | 132.28 |
| *OXR1* | | 62.75 |
| *SQSTM1* | | 19.86 |
| *CYGB* | | 15.7 |
| *MBL2* | | 11.13 |
| *NOX5* | | 10.53 |
| *HMOX1* | | 9.96 |
| *OXSR1* | | 6.09 |

**Table S3.** Immune regulatory PrimePCR targets with more than 4-fold deregulation in THP1 and PBMC.

| Target | THP1 (fold change) | PBMC (fold change) |
|---|---|---|
| *BIRC5* | 23.48 | 7.64 |
| *EGR1* | 7.97 | |
| *CRP* | 6.98 | |
| *PTGS2* | 6.52 | |
| *COL1A1* | 5.20 | |
| *CTGF* | 4.56 | |
| *NDFIP2* | 4.47 | |
| *KRAS* | 4.28 | |
| *MMP9* | -4.62 | |
| *VCAM1* | -4.98 | |
| *CCL2* | -5.19 | |
| *CXCL12* | -21.99 | |
| *IL18* | | 162.74 |
| *HLA-DRB1* | | 15.53 |
| *IL10* | | 15.53 |
| *MAPK3* | | 10.98 |
| *NFKBIA* | | 8.75 |
| *IL4* | | 7.69 |
| *TLR4* | | 7.03 |
| *UBB* | | 4.83 |
| *RELA* | | 4.18 |
| *COL1A1* | | 4.09 |

Supplemental Figures

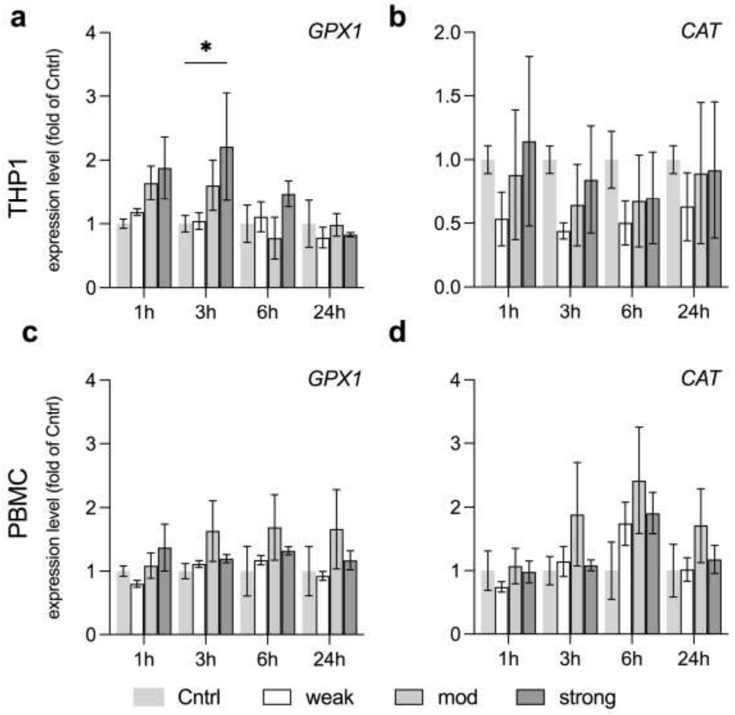


**Figure S1:** Expression levels of *GPX1* and *CAT* in THP1 (a, b) and PBMC (c, d) at different field strengths. *GPX1* levels increased in THP1 after 3 h of exposure to stEMF. No difference was found for *GPX1* in PBMC or *CAT* levels in both cell types. Data are given as fold of Cntrl (mean ± SEM, N = 3, n = 2). * $p < 0.05$ as indicated.
